# Supplementary material for: Are non-fungible payments attractive when they reduce risk exposure? Evidence from Colombia
Source: PLoS One. 2024 Jan 2;19(1):e0296299. doi: 10.1371/journal.pone.0296299 (PMC10760666; doi:10.1371/journal.pone.0296299)
Supplement: S1 Appendix — They can be found in the corresponding link. (PDF) [file pone.0296299.s001.pdf]

Online Appendix
Are non-fungible payments attractive when they reduce risk exposure? Evidence from Colombia

Alexander Cano[1], Darwin Cortés[2], César Mantilla*[2,3], Laura Prada[2], Medardo Restrepo[4]

1 Justus-Liebig-Universität Giessen, Giessen, Germany
2 Economics Department, Universidad del Rosario, Bogotá, Colombia
3 Loyola Behavioral Lab, Universidad Loyola Andalucía, Seville, Spain
4 Universidad del Quindío, Armenia, Colombia

The data and code to replicate these tables and figures, as well as those in the manuscript, can be found at the Open Science Framework platform: `https://osf.io/rp4xa/`.

A Additional Tables and Figures

Table A.1: Participants by municipality in the field sample

Municipality	Frequency	Percent	Order
Buenavista	20	9.35	Descending
Calarcá	23	10.75	Ascending
Circasia	19	8.88	Ascending/Descending
Córdoba	22	10.28	Ascending
Filandia	18	8.41	Descending
Génova	20	9.35	Ascending
La Tebaida	24	11.21	Ascending
Montenegro	20	9.35	Ascending
Pijao	20	9.35	Descending
Quimbaya	20	9.35	Descending
Salento	8	3.74	Descending

Table A.2: Balance test by treatment dimension - Field sample

	Order dimension			Fee dimension		
	Mean Ascending	Mean Descending	$p-$value	Mean Cash	Mean Voucher	$p-$value
Age	47.98	55.05	(0.002)	51.68	50.61	(0.646)
Women	0.33	0.34	(0.839)	0.35	0.32	(0.703)
Married	0.25	0.16	(0.081)	0.18	0.24	(0.329)
Income (log)	13.07	13.19	(0.130)	13.14	13.11	(0.721)
Payment in kind	0.32	0.25	(0.250)	0.27	0.31	(0.439)
Government's subsidy	0.27	0.31	(0.510)	0.31	0.27	(0.468)
Monthly salary	0.19	0.23	(0.543)	0.20	0.22	(0.759)
Elementary school	0.50	0.61	(0.095)	0.57	0.53	(0.604)
Household adults	2.90	2.64	(0.169)	2.78	2.78	(0.995)
Household kids	0.69	0.59	(0.497)	0.73	0.55	(0.181)
Siblings	4.05	4.14	(0.833)	4.20	3.97	(0.561)
Contributory scheme	0.17	0.28	(0.050)	0.22	0.22	(0.984)
Without old age plan	0.66	0.55	(0.105)	0.61	0.62	(0.840)
Willingness to take risks	5.66	6.19	(0.180)	5.83	5.96	(0.742)
Debts	0.51	0.42	(0.206)	0.50	0.44	(0.368)
Community support network	0.71	0.60	(0.104)	0.70	0.62	(0.192)
Land owning/leasing	0.34	0.40	(0.392)	0.39	0.34	(0.521)
Shocks	0.56	0.63	(0.334)	0.60	0.58	(0.820)
Health problems	0.45	0.56	(0.113)	0.54	0.47	(0.307)

Table A.3: Balance test by treatment dimension - Students sample

	Order dimension			Fee dimension		
	Mean Ascending	Mean Descending	$p-$value	Mean Cash	Mean Voucher	$p-$value
Age	24.97	24.03	(0.558)	23.91	25.09	(0.465)
Women	0.46	0.59	(0.283)	0.56	0.49	(0.550)
Married	0.11	0.06	(0.421)	0.06	0.11	(0.421)
Income(Log)	13.46	13.20	(0.295)	13.36	13.30	(0.806)
Government's subsidy	0.23	0.18	(0.597)	0.18	0.23	(0.597)
Monthly salary	0.43	0.53	(0.409)	0.47	0.49	(0.902)
Household adults	2.51	3.06	(0.069)	2.68	2.89	(0.490)
Household kids	0.49	0.53	(0.808)	0.47	0.54	(0.688)
Siblings	2.17	1.59	(0.081)	1.38	2.37	(0.002)
Contributory scheme	0.46	0.62	(0.187)	0.59	0.49	(0.401)
Without old age plan	0.66	0.56	(0.410)	0.68	0.54	(0.262)
Willingness to take risks	7.77	7.94	(0.625)	7.82	7.89	(0.858)
Debts	0.60	0.65	(0.692)	0.68	0.57	(0.375)
Community support network	0.69	0.62	(0.560)	0.65	0.66	(0.931)
Land owning/leasing	0.46	0.56	(0.406)	0.44	0.57	(0.286)
Shocks	0.51	0.62	(0.394)	0.62	0.51	(0.394)
Health problems	0.57	0.59	(0.890)	0.62	0.54	(0.536)

Table A.4: Uptake of the voucher option compared to 30 kCOP in cash.

	**Voucher value (in kCOP)**			
	$V = 25$	$V = 28$	$V = 30$	$V = 34$
**Panel I: Voucher uptake in the field sample**				
Field sample (N=214)	32.2	40.2	46.7	56.1
Show-up fee in voucher	27.6	39.0	43.8	56.2
Show-up fee in cash	36.7	41.3	49.5	56.0
χ^2 test for type of show-up fee [$p-$value]	[0.155]	[0.739]	[0.401]	[0.973]
Voucher values in descending order	41.7	46.9	52.1	59.4
Voucher values in ascending order	24.6	34.7	42.4	53.4
χ^2 test for order [$p-$value]	[0.008]	[0.072]	[0.157]	[0.380]
**Panel II: Voucher uptake in the students' sample**				
Students sample (N=69)	15.9	26.1	27.5	33.3
χ^2 test for field *versus* students' sample [$p-$value]	[0.009]	[0.035]	[0.005]	[0.001]
Show-up fee in voucher	17.1	34.3	40.0	45.7
Show-up fee in cash	14.7	17.6	14.7	20.6
χ^2 test for type of show-up fee [$p-$value]	[0.782]	[0.116]	[0.019]	[0.027]
Voucher values in descending order	14.7	20.6	23.5	20.6
Voucher values in ascending order	17.1	31.4	31.4	45.7
χ^2 test for ascending/descending order [$p-$value]	[0.782]	[0.305]	[0.463]	[0.027]

Table A.5: Treatment effects on the price of accepted vouchers

	Tobit		Ordered logit: Odd ratios	
	(1)	(2)	(3)	(4)
Show-up fee in voucher	-4.037**	-3.917**	0.374**	0.367**
	(1.943)	(1.879)	(0.151)	(0.148)
Field sample	-4.329*	0.339	0.339**	0.893
	(2.386)	(2.562)	(0.170)	(0.474)
Show-up fee in voucher × Field sample	4.527*	4.292**	3.285**	3.410***
	(2.325)	(2.077)	(1.739)	(1.560)
Descending order	-0.654	-0.633	1.116	1.108
	(1.900)	(1.832)	(0.415)	(0.406)
Descending order × Field sample	-1.235	-3.084	0.499	0.345**
	(2.206)	(2.058)	(0.244)	(0.159)
Formal employment deprivation		-0.358***		0.918**
		(0.127)		(0.0311)
Area (km2)		0.0215***		1.006***
		(0.00538)		(0.00172)
Average household size		9.304***		15.53**
		(3.267)		(17.82)
Constant	36.46***	31.01		
	(2.290)	(33.92)		
Test $\beta_{\text{Show-up fee}} + \beta_{\text{Show-up fee} \times \text{Field sample}}$	0.49	0.37	1.22	1.25
$[p-$value$]$	[0.693]	[0.636]	[0.552]	[0.293]
Observations	283	283	283	283

Additional controls: whether the session was the first or second in the day. Other municipal-level covariates that are non-significant: unsatisfied basic needs in the municipal seat, ratio of cultivated area over total area, average age, percentage of married population at the municipality level. Clustered standard errors in parentheses. *** p<0.01, ** p<0.05, * p<0.1.

Table A.6: Tobit model for the field sample with covariates of interest in an exploratory analysis.

	(1)	(2)	(3)
VARIABLES	Price of minimum accepted voucher		
Show-up fee in voucher	0.283	0.317	0.358
	(0.745)	(0.716)	(0.733)
Descending order	-3.432***	-3.337***	-3.174***
	(0.854)	(0.963)	(0.967)
Willingness to take risks		-0.302**	-0.381***
		(0.127)	(0.129)
Agricultural laborer		1.569	1.453
		(0.994)	(0.940)
Women		-1.478**	-0.946
		(0.729)	(0.829)
Age		-0.0263	-0.0115
		(0.0331)	(0.0327)
Primary school or less		0.674	0.649
		(1.086)	(1.111)
Married		-0.109	-0.167
		(1.092)	(1.080)
Government's subsidy			-2.346***
			(0.788)
Monthly salary			-1.363
			(1.049)
Payment-in-kind			0.107
			(0.799)
Constant	34.14	15.52	-2.979
	(35.08)	(30.69)	(32.80)
Observations	214	212	212

All regressions included municipal-level covariates and a dummy for session order within a municipality. Standard errors clustered at the session level in parentheses. *** p<0.01, ** p<0.05, * p<0.1

B Experimental Protocol: Translated Version

General instructions

Welcome. We appreciate that you accepted the invitation to this activity that will last around 60 minutes. This time includes the explanation of the activity, the time in which you make your decisions, and a short survey. In this activity, your decisions have economic consequences to make them more similar to the decisions you make in your daily life. Winnings from this game are not paid for participating, so we hope that you will participate in future activities of other researchers, even if no winnings are involved.

The funds to cover these expenses have been provided by [undisclosed institution] within the framework of a project on labor markets financed by [undisclosed funding source] The information collected will be anonymized and only those responsible for the project will have access to it.

Initial profit for participating

Just for participating, answering a survey and staying until the end you will win $10,000. This money will be given to you *[in cash/vouchers, whose redemption at a nearby supermarket will be explained below].*

Additionally, you will complete a task that will take between 5 and 10 minutes. You will be able to choose between two payment schemes for carrying out this task.

`>>In the treatment with show-up fee paid as a voucher, display the voucher.<<`

Pay in cash or vouchers?

We want to understand if you prefer cash or voucher payment.

You can exchange the vouchers after finishing the activity for food or toiletries at the supermarket [**NAME OF SUPERMARKET,**] which is located [**DESCRIPTION IN TIME/DISTANCE ON HOW TO GET THERE**].

At the end of the activity we will throw a die that has 4 green faces, 1 red face and 1 white face.

`>>Show dice and their respective faces.<<`

If you receive your winnings in cash, the roll of the dice is the only thing that determines your winnings. If it lands on the red side, it will cause you to lose $20,000 of your winnings.

If you receive your winnings as tokens, you will roll the dice first. If the die lands on the red side, you will be able to flip a coin that works as “insurance”.

If the coin lands on the green side, it will prevent you from losing $20,000 of your winnings. In other words, receiving the winnings in vouchers halves your chances of losing that $20,000. The white side of the die has no function. If the white face comes up when rolling the die, the die will be rolled again until a green or red face comes up. Payment in vouchers can only be spent on food or toiletries at the supermarket, but in return you have an “insurance”, which reduces the chances that a negative event will reduce your earnings by $20,000.

Since we are interested in how much money in vouchers makes this option interesting to you, we will make the decision to keep cash or vouchers four times. The amount offered as vouchers will change, while the cash payment will be fixed. In the end, we will choose one of four random decisions to calculate your payouts.

Now we are going to explain what is the task that you will have to carry out in order to receive the earnings of the activity.

Explanation of the separate bean task

You will receive a bag of beans and two small buckets. You must separate the red grains, which will go into the small bucket, from the white grains, which you will leave in the big bucket.

We will indicate the level until which the small buckets should be filled. You will need to complete this task in order to receive any earnings from the activity. It doesn't matter how long it takes you to complete it, but it's important that you do it as quickly as possible because we won't be able to continue until everyone has finished. We will measure the time it takes you to complete the task, but this will not affect your earnings.

`>>Show the buckets and the tape or line to which they are expected to be filled.<<`

Explanation of the decision-making process

Now we are going to explain in detail how the decision-making will be. You have received a plastic card with the number 1, this means that with this card you will make the Decision #1.

`>>Show the card with Decision #1 .<<`

(this card is similar to the left panel of Figure ??)

If you choose CASH, you can win $30,000 if one of the 4 green sides of the die comes up, or $10,000 if the red side of the die comes up. If you choose the VOUCHERS, you can win $34,000 if one of the 4 green faces of the dice comes up. If the red side of the die comes up, you have an “insurance” that will allow you to flip the coin. If the coin lands

on the green side, you will win $34,000. If the coin lands on the red side, you will win $14,000. After making this decision, you can remove the other three laminated cards from the envelope, as we indicate.

Summary of instructions

This poster summarizes the stages of the activity.

```
>>Show the poster and explain each stage of the activity.<<
```

(the poster corresponds to the right panel of Figure ??)

First, we are going to read and sign the Informed Consent. This is a document in which you declare that you are here at your will, that you have understood the instructions of the activity, and that you will comply with the biosafety instructions. In exchange, we declare that the data will be used confidentially and for purely academic purposes, and that we will make the promised payments.

Then, we are going to make the four decisions. Remember that they are very similar decisions, except that the amount we offer you in VOUCHERS will change, whereas the amount in CASH will be fixed. Once you make all four decisions, you will do the task of separating coffee beans.

One you take all the decisions and complete the task, you will draw one ball corresponding to the decision number we will pay to you. Once we have the selected decision, we will roll the dice and flip the coin to compute your payment. Finally, while we calculate your earnings and prepare the receipt for you to sign, we will ask you to complete a survey.

Reading and signing the informed consent

```
>>Ask participants to remove the informed consent form from the envelope.
Read the consent aloud and ask them to sign it.
Check that all participants signed.<<
```

Making the first decision

Now that we've signed informed consent, let's quickly review the decision-making process.

```
>>Ask participants to remove card number 1 from the envelope.
Check in each decision that the card outside corresponds to the decision
announced out loud.<<
```

You have received a plastic card to make Decision #1. If you choose CASH, you can win $30,000 if one of the 4 green sides of the die comes up, or $10,000 if the red side of the die comes up. If you choose the VOUCHERS, you can win $34,000 if one of the 4 green faces of the dice comes up. If the red side of the die comes up, you have an "insurance" that will allow you to flip the coin. If the coin lands on the green side, you will win $34,000. If the coin lands on the red side, you will win $14,000.

Please use the marker to make an "X" on the option you prefer, CASH or VOUCHERS. Once all the participants keep their marked card in the envelope, we will move on to the second decision.

```
>>Collect card #1 and fill out the session registration.<<
```

Making the second to fourth decisions

```
>>Read again the consequences of choosing cash or voucher.
The script for decision making #2 through #4 can be a bit repetitive.
It can be shortened if it is considered that the participants understood after the
first decision.
Pick up the corresponding card and fill out the session registration.<<
```

Performing the bean separation task

You have already made all four decisions. Now we are going to give you the bag with beans and the two buckets. Please do not start until we tell you to. Remember to separate the red grains, which should go into the small bucket, from the white grains. For this, you will not be able to spread the beans on the table/chair you are on.

You will need to complete the task to receive the earnings from the activity. No matter how long it takes you to complete it, but we won't be able to continue until everyone has finished. We will measure the time it takes you to complete the task, but this will not affect your earnings.

Payment computation: choice selection, dice roll and coin toss

We will now decide which of the four tasks will be used to calculate the earnings for each participant. The monitor will approach you, and you will draw a ball from the bag. Each ball is marked with a number from 1 to 4. The number you roll will correspond to the decision that we will pay you.

After having defined the decision that will be paid, you will roll the dice. If the red side of the die comes up and you chose the CASH payout for that decision, you will lose $20,000 of your payout. If the red side of the die lands and you chose the VOUCHER

payout for that decision, you will flip a coin. Only if the coin also lands on the red side will you lose $20,000 of your payout. If the white face lands when the die is rolled, it will be repeated until the roll lands a green or red face.

Survey and final payments

While we finish calculating your earnings, one of the monitors will help you filling a survey. We will ask you for characteristics about yourself, your work and your home. Remember that all information collected within this activity, including your responses to the survey, will be used confidentially and solely for the purposes of this research.

Voucher redemption

You have received the payment for the activity in a voucher. You can redeem it for food or toiletries at the supermarket [**NAME OF SUPERMARKET.**]

Once you have decided how to spend the vouchers, you can approach one of the activity monitors in the supermarket, hand over the vouchers, and claim the desired items. If you want to spend some of the initial money you received for your participation in the store, you can do that too.

C Experimental Protocol: Original (Spanish) Version

Instrucciones generales

Bienvenidos. Agradecemos que aceptaran la invitación a esta actividad que durará alrededor de 60 minutos. Este tiempo incluye la explicación de la actividad, la toma de sus decisiones, y una breve encuesta. En esta actividad sus decisiones tienen consecuencias económicas, de modo que sean más parecidas a las decisiones que toma en su vida diaria. Las ganancias que usted se lleve en este juego no corresponden a un pago por participar, por lo que esperamos que participe en futuras actividades de otros investigadores, aún si no hay ganancias de por medio.

Los fondos para cubrir estos gastos han sido proporcionados por [institución no revelada] en el marco de un proyecto sobre mercados laborales financiado por [financiador no revelado] La información recolectada será anonimizada y solo los responsables del proyecto tendrán acceso a ella.

Ganancia inicial por participar

Por el sólo hecho de participar, responder una encuesta y quedarse hasta el final usted ganará $10.000. Este dinero se le entregará *[en efectivo/en vales, cuyo canje en un supermercado cercano se explicará a continuación].*

Adicionalmente, usted hará una tarea que le tomará entre 5 y 10 minutos. Usted podrá escoger entre dos esquemas de pago por la realización de esta tarea.

`>>Display the voucher in the treatment with show-up fee paid in voucher.<<`

¿Pago en efectivo o en vales?

Queremos entender si usted prefiere un pago en efectivo o en vales.

Los vales los podrá intercambiar tras finalizar la actividad por alimentos o artículos de aseo en el supermercado [**NOMBRE DEL SUPERMERCADO,**] que está ubicado [**DESCRIPCIÓN EN TIEMPO/DISTANCIA SOBRE CÓMO LLEGAR**].

Al final de la actividad lanzaremos un dado que tiene 4 caras verdes, 1 cara roja y 1 cara blanca.

`>>Mostrar dado y sus respectivas caras.<<`

Si usted recibe sus ganancias en efectivo, el lanzamiento del dado es lo único que determina sus ganancias. Si cae por la cara roja, hará que usted pierda $20.000 de sus ganancias.

Si usted recibe sus ganancias como vales, usted lanzará primero el dado. Si el dado cae por la cara roja, usted podrá lanzar una moneda que funciona como un "seguro".

Si la moneda cae por la cara verde, evitará que usted pierda $20.000 de sus ganancias. Dicho de otro modo, recibir las ganancias en vales reduce a la mitad sus chances de perder esos $20.000. La cara blanca del dado no tiene ninguna función. Si cae la cara blanca al lanzar el dado, se lanzará de nuevo el dado hasta que caiga una cara verde o roja. El pago en vales sólo lo puede gastar en alimentos o artículos de aseo en el supermercado, pero a cambio tiene el "seguro", que reduce los chances de que un evento negativo reduzca sus ganancias en $20.000.

Como nos interesa saber qué cantidad de dinero en vales hace que esta opción sea interesante para usted, tomaremos cuatro veces la decisión de quedarse con el efectivo o con los vales. El monto ofrecido como vales irá cambiando, mientras el pago en efectivo será fijo. Al final, escogeremos una de las cuatro decisiones al azar para calcular sus pagos.

Ahora vamos a explicar cuál es la tarea que deberán realizar para poder recibir las ganancias de la actividad.

Explicación de la tarea de separar frijol

Usted va a recibir una bolsa con granos de frijol y dos pequeños baldes. Usted debe separar los granos rojos, que irán al balde pequeños, de los granos blancos, que dejará en el balde grande.

A continuación le indicaremos hasta dónde deben ser llenados los baldes pequeños. Usted deberá completar esta tarea para poder recibir las ganancias de la actividad. No importa cuánto tiempo le tome completarla, pero es importante que la realice lo más rápido posible porque no podremos continuar hasta que todos hayan terminado. Nosotros mediremos el tiempo que le tome terminar la tarea, pero esto no afectará sus ganancias.

`>>Mostrar los baldes y la cinta o la línea hasta la cual se espera que sean llenados.<<`

Explicación de la toma de decisiones

Ahora vamos a explicar en detalle cómo será la toma de decisiones. Usted ha recibido una tarjeta plastificada con el número 1, esto quiere decir que con esta tarjeta tomará la Decisión #1.

`>>Mostrar la tarjeta con la Decisión #1 .<<`

(this card is similar to the left panel of Figure ??)

Si usted escoge el EFECTIVO, puede ganar $30.000 si sale una de las 4 caras verdes del dado, o $10.000 si sale la cara roja del dado. Si usted escoge los VALES, puede ganar $34.000 si sale una de las 4 caras verdes del dado. Si sale la cara roja del dado, usted tiene el "seguro" que le permitirá lanzar la moneda. Si la moneda cae por la cara verde, ganará $34.000. Si la moneda cae por la cara roja, ganará $14.000. Luego de tomar esta decisión usted podrá sacar del sobre las otras tres tarjetas plastificadas, según le vayamos indicando.

Resumen de las instrucciones

Este afiche resume las etapas de la actividad.

```
>>Mostrar el afiche y explicar cada etapa de la actividad.<<
```

(the poster corresponds to the right panel of Figure ??)

Primero, vamos a leer y firmar el Consentimiento Informado. Este es un documento en el que ustedes declaran que están aquí bajo su voluntad, que han entendido las instrucciones del ejercicio, y que cumplirán con las instrucciones de bioseguridad. A cambio, nosotros declaramos que los datos serán utilizados de forma confidencial y con fines puramente académicos, y que realizaremos los pagos prometidos.

Después, vamos a tomar las cuatro decisiones. Recuerde que son decisiones muy parecidas, excepto que el monto que le ofrecemos en VALES irá cambiando, y el monto en EFECTIVO estará fijo. Una vez tome las cuatro decisiones, usted hará la tarea de separar granos de café.

Una vez usted tome las decisiones y complete la tarea, usted sacará una bola cuyo número corresponderá a la decisión con la que calcularemos su pago. Una vez seleccionada la decisión que le pagaremos, realizaremos el lanzamiento del dado y de la moneda para calcular sus ganancias. Finalmente, mientras calculamos sus ganancias y preparamos el recibo que deberá firmar, le pediremos que responda una encuesta.

Lectura y firma del consentimiento informado

```
>>Pedir que saquen el consentimiento informado del sobre.
Leer el consentimiento en voz alta y pedir que lo firmen.
Revisar que todos los participantes firmaron.<<
```

Toma de la primera decisión

Ahora que hemos firmado el consentimiento informado repasemos rápidamente la toma de decisiones.

>>Pedir que saquen la tarjeta numero 1 del sobre.
Revisar en cada decisión que la tarjeta afuera sea la correspondiente a la decisión anunciada en voz alta.<<

Usted ha recibido una tarjeta plastificada para tomar la Decisión #1. Si usted escoge el EFECTIVO, puede ganar $30.000 si sale una de las 4 caras verdes del dado, o $10.000 si sale la cara roja del dado. Si usted escoge los VALES, puede ganar $34.000 si sale una de las 4 caras verdes del dado. Si sale la cara roja del dado, usted tiene el "seguro" que le permitirá lanzar la moneda. Si la moneda cae por la cara verde, ganará $34.000. Si la moneda cae por la cara roja, ganará $14.000.

Por favor use el marcador para hacer una "X" en la opción que prefiere, el EFECTIVO o los VALES. Una vez todos los participantes guarden en el sobre su tarjeta marcada, pasaremos a la segunda decisión.

>>Recoger la tarjeta #1 y diligenciar el registro de la sesión.<<

Toma de la segunda a cuarta decisión

>>Leer de nuevo las consecuencias de escoger el efectivo o el vale.
El libreto para la toma de decisiones #2 hasta #4 puede ser un poco repetitivo.
Es posible acortarlo si se considera que los participantes entienden con frases más cortas después de la primera instrucción.
Recoger la tarjeta correspondiente y diligenciar el registro de la sesión.<<

Realización de la tarea de separar frijol

Usted ya tomó las cuatro decisiones. Ahora vamos a entregarle la bolsa con granos de frijol y los dos baldes. Por favor no comience hasta que se lo indiquemos. Recuerde que debe separar los granos rojos, que deberán ir al balde pequeño, de los granos blancos. Para esto, usted no podrá regar los granos de frijol sobre la mesa/silla sobre la que se encuentra.

Usted deberá completar la tarea para recibir las ganancias de la actividad. No importa cuánto tiempo le tome completarla, pero no podremos continuar hasta que todos hayan terminado. Nosotros mediremos el tiempo que le tome terminar la tarea, pero esto no afectará sus ganancias.

Elección de la decisión a pagar, lanzamiento del dado y la moneda

Ahora decidiremos cuál de las cuatro tareas se utilizará para calcular las ganancias de cada uno. El monitor se acercará a usted, y usted sacará una bola de la bolsa. Cada bola

está marcada con un número del 1 al 4. El número que saque corresponderá a la decisión que le pagaremos.

Tras haber definido la decisión que será pagada, usted lanzará el dado. Si cae la cara roja del dado y usted eligió el pago en EFECTIVO para esa decisión, usted perderá $20.000 de su pago. Si cae la cara roja del dado y usted eligió el pago en VALES para esa decisión, usted lanzará una moneda. Sólo si la moneda también cae por la cara roja usted perderá $20.000 de su pago. Si cae la cara blanca al lanzar el dado, se repetirá hasta el lanzamiento que caiga una cara verde o roja.

Encuesta y pagos finales

Mientras terminamos de calcular sus ganancias, uno de los monitores le realizará una encuesta. Le preguntaremos por características sobre usted, su trabajo y su hogar. Recuerde que toda la información recopilada dentro de esta actividad, incluyendo sus respuestas en la encuesta, serán utilizadas de manera confidencial y únicamente con fines de esta investigación.

Canje de los vales

Usted ha recibido el pago de la actividad en vales. Usted podrá cambiarlos por alimentos o artículos de aseo en el supermercado [**NOMBRE DEL SUPERMERCADO.**]

Una vez haya decidido cómo gastar los vales, usted podrá acercarse a uno de los monitores de la actividad que se encuentren en el supermercado, entregarle los vales, y reclamar los artículos deseados. Si usted desea gastar parte del dinero inicial que recibió por su participación en la tienda, también podrá hacerlo.
