# Supplementary material for: Are non-fungible payments attractive when they reduce risk exposure? Evidence from Colombia
Source: PLoS One. 2024 Jan 2;19(1):e0296299. doi: 10.1371/journal.pone.0296299 (PMC10760666; doi:10.1371/journal.pone.0296299)
Supplement: Attachment — Submitted filename: Response letter_RR.pdf [file pone.0296299.s002.pdf]

Editor Comments:

The experimental design is carefully thought out and well sampled. The study is interesting and worthy of being an experiment to test financial tolerance in developing countries.

Dear Editor,

Many thanks for the opportunity to revise our manuscript. We believe that the comments provided by the two referees and by you were very helpful in improving our paper. The required amendments to the voucher values' explanation (and their connection with the hypotheses and departures from the expected utility model) were very useful. Similarly, we paid more attention to the explanation of our procedure and some specific details about the fieldwork (e.g., how we determined the show-up fee). We also followed the Reviewers' advice regarding the display of results (we sent the main results table to the Appendix and replaced it with a figure that summarizes our findings), and the separation of the results from the discussion (which we also connected better with the existing literature).

Below, you will find in blue our responses to each one of the comments. Direct quotes from the paper are indented and displayed in a different font.

Many thanks again. We are looking forward to any further requests or clarification.

However, for the benefit of the reader, please add the following points:

1) Please detail the progression procedure step-by-step, as it may be misleading for MLPs. Also, please explain in detail how to determine the show-up fee.

Thank you for this comment. We now provide a more detailed progression procedure in the new subsection entitled "Timing of a session". Here, we go step by step on the execution of our sessions. We took the existing structure and transformed it into the following list:

(i) Introduction
(ii) Introduction to the four decisions
(iii) Introduction to the real-effort task
(iv) Detailed explanation of decisions
(v) Getting consent
(vi) Decision-making
(vii) Completion of the real-effort task
(viii) Draw of payment decision
(ix) Questionnaire

All these steps are explained in detail in lines 263-343. We omit their description in this letter to not make it too large, and because most of the text was already written as you may check with the tracked changes version.

Regarding the explanation of the show-up fee payment, we added a short explanation in lines 210-214. The new text is underlined in the following sentence:

> At the beginning of each session, we informed participants that just for coming and staying until the end of the activity, they would receive 10 kCOP. This show-up fee offers a balance between a fixed payment sufficiently low to preserve the salience of the decision-based payments but sufficiently high to guarantee a minimum compensation of 20 kCOP for participants facing the negative shock.

2) Why is the expected utility hypothesis not valid? Why can't people's behavior during the experiment be explained by the certainty equivalent in expected utility as presented in the classical insurance model? Discuss this in detail. Or use prior research to explain this in your intro.

Thank you for raising this point. We have now elaborated the discussion on the expected utility (EU) hypothesis in the introduction and in the experimental design section. We now made clear that, as the voucher value increases, the EU hypothesis falls short explaining the choice of cash over voucher payments, giving room to non-fungibility to operate.

In the introduction, we added this discussion in lines 38-46. It reads as follows:

> The cash payment is the same across the four decisions, but we vary the voucher payment. We start with a voucher whose expected value is lower than the cash expected payment. This decision helps understand whether the risk reduction is sufficiently appealing and whether participants are willing to choose this payment method, implicitly revealing that they treat the voucher as fungible. As the voucher's expected value increases in the subsequent decisions, the expected utility model falls short in explaining why participants do not opt for the less risky and better-paid option, revealing that the violations of fungibility make the voucher payment unattractive for some participants.

In the section devoted to the experimental design, we added a new Table 1. The objective of this new Table 1 was to display much earlier the relevant elements from the experimental design in terms of expected payoffs (previously Panel A in the old and removed version of Table 1). At the bottom of Table 1, we also added a couple of lines on the predictions under expected utility with fungibility. This table looks as follows:

Table 1. Comparison, in expected values, between the *Cash* and *Voucher* payment methods

	Voucher value (in kCOP)			
	$V = 25$	$V = 28$	$V = 30$	$V = 34$
E(Voucher)	23	26	28	32
E(Cash)	26	26	26	26
E(Voucher) $-$ E(Cash)	-3	0	2	6
E(Voucher)/E(Cash)	0.88	1	1.07	1.23
Payment Voucher/Cash (pre-shock)	0.83	0.93	1	1.13
**Predictions under expected utility**				
Risk-neutrality and fungibility	Cash	Indifference	Voucher	Voucher
Slight risk-aversion and fungibility	Cash	Voucher	Voucher	Voucher

The introduction to this Table, in lines 131-137 reads:

To explain better the rationale behind the specific values of V, let us introduce the expected values of the cash and voucher payments in Table 1. By design, E(Voucher), the expected value of the voucher, is increasing in V. By contrast, and to make the decisions more tractable, the cash payment and its expected value, E(Cash), are held constant. The three rows in the middle of this table provide a direct comparison between the expected payoffs: their difference, their ratio, and their ratio ignoring the potential losses (i.e., the ratio before the chance of losing 20 KCOP).

We then explain the rationale behind each one of the voucher values, V. Lines 138-172 read:

Let us start with $V = 25$, the lowest voucher value and, therefore, the less attractive with respect to the cash payment. In terms of predictions, it should only be selected by participants that are sufficiently risk averse to value the reduction in risk exposure *and* see the voucher as fungible. Our choice of $V = 25$, instead of any other $V < 28$ that also yielded E(Voucher)/ E(Cash) <1, considered the transaction costs of lowering risk exposure. In fact, the payment ratio 25/30 (or 83% in the table) resembles the contribution rates to social security in the Colombian labor market, which are about 17% of the income. An alternative interpretation is that, if the risk exposure is directly covered by rural cooperatives, this could be a transaction fee charged by the cooperative for managing this additional risk. More generally, this scenario yields an idea of the acceptance rate of this risk-reduction policy if the users pay the costs associated with the risk transfer as an insurance premium.

When $V = 28$, we have E(Voucher)/E(Cash)=1. Having the same expected value, and a lower risk exposure with the voucher payment, any degree of risk aversion would be sufficient to prefer the voucher if the payment methods are treated as fungible. Given the prevalence of risk-aversion [30], low to intermediate take-up levels of the voucher payments for $V = 28$ would be more likely to signal the presence of non-fungibility than a disproportionate number of risk-seeking participants.

By setting $V = 30$ in one of our decisions, we can study the choice of payment method while keeping the same initial paid amount (i.e., before the potential loss). This decision is illustrative because it makes the comparison of expected losses very straightforward: both choices pay the same and face a loss of the same magnitude, but the probability of this loss is twice with one payment method with respect to the other. Hence, the trade-off between risk exposure and non-fungibility is much clearer. Choosing the cash over the voucher payment would be, in this case, a violation of fungibility. As an alternative but non-mutually exclusive interpretation, the difference in payment methods would be sufficiently salient to induce a problem of probability neglect [35], where participants are not sensitive to the absolute risk reduction to avoid the non-fungible payment.

Finally, the decision with $V = 34$ yields a larger voucher payment, both *ex ante* (13%) and in expected value (23%), compared to cash. We set up this relatively large V aiming to find a reasonable subsidy amount that would guarantee almost complete acceptability of these non-fungible payments. A voucher acceptance rate far from 100% would suggest that even if the risk-transfer mechanism is externally funded, non-fungibility remains a threat to its popularity.

And we close this added explanation with the description of the bottom section in Table 1. Lines 173-178 read:

The bottom of Table 1 summarizes the predictions under the expected utility model for each V. Note that, if risk neutrality and fungibility hold, we should observe the choice of cash payments only for $V \leq$

28. If risk neutrality is replaced by mild risk aversion (to break the indifference from a lower risk exposure with the same expected value), we should observe cash payments only for $V = 25$. In the discussion section, we will analyze our findings in the light of alternative behavior models.

Reviewer #1

"Non-fungibility reduces the attractiveness of risk-reducing payments among rural workers: a lab-in-the-field experiment in Colombia."

Dear Reviewer,

Many thanks for all your comments. We consider that they helped us to address with more clarity some of the messages we wanted to convey. For instance, the more thorough discussion on the rationale for the different voucher values and the expanded discussion on the display order of the vouchers stem from your suggestions. Thanks again.

1. The title is descriptive but might be too lengthy for quick comprehension. Consider shortening the title while retaining its essence, e.g., "Non-fungibility and Risk Preferences: A Field Experiment in Colombia."

Thank you for pointing out that the title was quite lengthy. We now changed it to "Are non-fungible payments attractive when they reduce risk exposure? Evidence from Colombia."

Two notes on why we chose an alternative shorter title with respect to your proposal.

First, by evoking risk preferences, other scholars using similar methods that read this paper would expect incentivized measurements of risk aversion and ours was non-incentivized (though based on the Dohmen et al.'s instrument which has been shown to be correlated with incentivized lotteries). Hence, we talk about the reduction in risk exposure (a property of our decision environment) rather than about risk preferences.

Given this discussion, we also added lines 321-327 to introduce much earlier the explanation of our non-incentivized measure of risk attitudes. They read:

> We administered a survey to collect information regarding the participant's demographics, access to credit, and response to health shocks. This survey included a non-incentivized measurement of willingness to take risks by Dohmen et al. [11], who showed that responses are correlated with the choice of more risky incentivized lotteries. We employed this non-incentivized question to reduce the cognitive burden from our instrument and avoid further steps in the payoff computations, which may confuse participants.

Second, we preferred not to mention "Field Experiment" in the title because the first line of the abstract clarifies this (as well as the rural nature of our sample). Also, because "Field Experiment" may evoke a randomized controlled trial, causing confusion to some readers.

2. The abstract provides a clear overview but lacks specific results or statistical significance indicators. Include key statistical findings in the abstract to give readers immediate insight into the study's outcomes.

Thanks. We have adjusted the abstract and provided p-values of statistical comparisons between samples and between the ascending and descending order within the sample of rural workers,

which are the two comparative statements made in the abstract. We underlined the new content on statistical reports in the following version of the updated abstract:

> We conducted a lab-in-the-field experiment in which 214 Colombian rural workers had to choose between cash or a voucher payment for completing a real-effort task. Although the voucher may be perceived as non-fungible, it halves the probability of suffering a negative shock that will reduce the participant's payment by two-thirds. Participants make four decisions in which we vary the voucher values such that this payment method offers, in expectation, between 88% to 123% of the cash payment (fixed across decisions). We find that take-up rates go from 32% to 56%, from the least to the more generous voucher. These rates are consistently larger compared to a reference sample of undergrad students from the same region (*p*-values from the χ^2 tests for all four decisions fall below 0.035). Our between-subjects variations reveal that presenting the vouchers in descending order yields a higher take-up than the ascending order (*p*<0.001 for the corresponding coefficient in a tobit and ordered logit regressions including municipality characteristics, an effect driven by the two decisions with the lowest voucher values, with *p*-values of 0.008 and 0.072 in the χ^2 tests, respectively). We interpret this result as an endowment effect of the voucher's risk reduction.

3. While the choice between cash and voucher is clear, the rationale behind the specific voucher values and their expected outcomes is not elaborated upon. Provide a theoretical framework or prior research that guided the choice of voucher values. Discuss the expected behaviour based on economic or psychological theories.

Thank you for this suggestion that helped us improve the exposition of our design choices. We rewrote the first part of the Experimental Design to provide a clearer rationale for the specific voucher values. It appears now under the subsection's title "Set up of decisions between cash and voucher payment". In this new subsection, we added a new Table 1 (which borrowed elements from Panel A in the previous Table 1) and complemented it with the predictions under the expected utility model and the assumption of fungibility. The added text (lines 125-178) reads:

> Participants took four decisions with different voucher values given by $V \in \{25;\ 28;\ 30;\ 34\}$ [kCOP] (in USD, V goes from 6.45 to 8.77). Our reference point for creating the trade-offs between payment methods was the cash payment of 30 kCOP. This value corresponded to the standard daily wage of rural work (*jornal*) in the time and region of our study. This amount was attractive and compensated for the participants' opportunity cost of time.
>
> To explain better the rationale behind the specific values of V, let us introduce the expected values of the cash and voucher payments in Table 1. By design, E(Voucher), the expected value of the voucher, is increasing in V. By contrast, and to make the decisions more tractable, the cash payment and its expected value, E(Cash), are held constant. The three rows in the middle of this table provide a direct comparison between the expected payoffs: their difference, their ratio, and their ratio ignoring the potential losses (i.e., the ratio before the chance of losing 20 KCOP).
>
> Let us start with $V = 25$, the lowest voucher value and, therefore, the less attractive with respect to the cash payment. In terms of predictions, it should only be selected by participants that are sufficiently risk averse to value the reduction in risk exposure *and* see the voucher as fungible. Our choice of $V = 25$, instead of any other $V < 28$ that also yielded E(Voucher)/ E(Cash) <1, considered the transaction costs

of lowering risk exposure. In fact, the payment ratio 25/30 (or 83% in the table) resembles the contribution rates to social security in the Colombian labor market, which are about 17% of the income. An alternative interpretation is that, if the risk exposure is directly covered by rural cooperatives, this could be a transaction fee charged by the cooperative for managing this additional risk. More generally, this scenario yields an idea of the acceptance rate of this risk-reduction policy if the users pay the costs associated with the risk transfer as an insurance premium.

When $V = 28$, we have E(Voucher)/E(Cash)=1. Having the same expected value, and a lower risk exposure with the voucher payment, any degree of risk aversion would be sufficient to prefer the voucher if the payment methods are treated as fungible. Given the prevalence of risk-aversion [30], low to intermediate take-up levels of the voucher payments for $V = 28$ would be more likely to signal the presence of non-fungibility than a disproportionate number of risk-seeking participants.

By setting $V = 30$ in one of our decisions, we can study the choice of payment method while keeping the same initial paid amount (i.e., before the potential loss). This decision is illustrative because it makes the comparison of expected losses very straightforward: both choices pay the same and face a loss of the same magnitude, but the probability of this loss is twice with one payment method with respect to the other. Hence, the trade-off between risk exposure and non-fungibility is much clearer. Choosing the cash over the voucher payment would be, in this case, a violation of fungibility. As an alternative but non-mutually exclusive interpretation, the difference in payment methods would be sufficiently salient to induce a problem of probability neglect [35], where participants are not sensitive to the absolute risk reduction to avoid the non-fungible payment.

Finally, the decision with $V = 34$ yields a larger voucher payment, both *ex ante* (13%) and in expected value (23%), compared to cash. We set up this relatively large V aiming to find a reasonable subsidy amount that would guarantee almost complete acceptability of these non-fungible payments. A voucher acceptance rate far from 100% would suggest that even if the risk-transfer mechanism is externally funded, non-fungibility remains a threat to its popularity.

The bottom of Table 1 summarizes the predictions under the expected utility model for each V. Note that, if risk neutrality and fungibility hold, we should observe the choice of cash payments only for $V \leq 28$. If risk neutrality is replaced by mild risk aversion (to break the indifference from a lower risk exposure with the same expected value), we should observe cash payments only for $V = 25$. In the discussion section, we will analyze our findings in the light of alternative behavior models.

4. The comparison with undergrad students might not be directly relevant or might lack context. Elaborate on the reason for this specific comparison. If it's to show a difference in risk preferences based on demographics, then more demographic data should be provided.

Thanks. This is an important point that we now address in our manuscript with two major modifications.

First, we reorganized the preview of results introduction by request of Reviewer 2, and we now have a paragraph comparing rural workers with students. As part of this paragraph, we summarized our main purpose for replicating the protocol with a sample of students, which is the fact that vouchers would be in theory perceived as less fungible by students because, we infer

from their household's composition, that they are not in charge of grocery shopping. Lines 74-79 read:

> To put the voucher acceptability in perspective, we implemented the same instrument with a sample of 69 university students from the region's main city, Armenia. Non-fungibility should be even starker in this sample since students in this region are rarely in charge of grocery shopping at their homes. Indeed, we find that their voucher take-up goes from 16% to 33%, from the least to the most generous voucher.

Second, in the Sampling section, we provide further details and elaborate on the reasons for including the sample of students with additional demographic information. As lines 365-378 read, the main reason is that we conjectured they would perceive the voucher as less fungible due to the abovementioned reasons. A second reason, valid prior to data analysis, is that the students' sample may offer some insights related to the complexity of instructions in case the rural sample was not responding to changes in the voucher value, V.

> We conducted ten additional sessions with a total of sixty-nine students in Armenia, the capital city of Quindío, in September 2021. We collected the data on this additional sample with two comparative purposes. First, students were, on average, 24.5 years old (std. dev. 6.6), and they were rarely in charge of grocery shopping. The reason is that they usually live with their elders in this city or nearby municipalities (the average number of adults in their household is 2.8, only 8.7% reported being married or in cohabitation, and 38% reported receiving a monthly allowance). Hence, we hypothesize that students would treat the voucher as less fungible than the rural sample. Given these conditions of the students' sample, they serve as a benchmark comparison for the voucher acceptability among rural workers, regardless of V. Second, lab-in-the-field studies are subject to concerns about whether the protocol was clear for participants. Replicating the protocol with a students' sample is *ex ante* helpful to validate, in case of finding an unexpected behavioral pattern in the sample of interest (e.g., lack of sensibility to V), if it may be caused by confusion with the protocol.

5. The finding about the order of voucher presentation is interesting but lacks a deeper exploration. Delve into the psychological or behavioural reasons behind this finding. Consider referencing literature on choice architecture or framing effects.

Thanks. We created a discussion section where we wrote a more detailed exploration of our findings regarding the order of the voucher presentation. As suggested, we add some references to this discussion. It turned out that we found a couple of papers directly discussing the endowment effect of risk, and one of them was also a lab-in-the-field experiment conducted in a developing country. The relevant lines 600-617, added to the new discussion section, read:

> Rural workers took the vouchers more often when they were presented in descending order. In line with the hypothesis described at the end of the Experimental Design section, one explanation for this result is that the partial insurance from the voucher payment appears to trigger an endowment effect that maintains the attractiveness of this payment method in the subsequent decisions with lower voucher values. In other words, rural workers assign more value to the voucher because of the implicit insurance it brings. Hence, when the value of the voucher decreases in further decisions, it is less likely that these workers will switch to the cash option because they value more the implicit risk reduction compared to

> the participants who took the decisions in ascending order and did not benefit from the lowest risk exposure. This endowment effect for risk has been documented, with an emphasis on the differences in the predicted behavior when the reference point is stochastic [34]. More importantly, lab-in-the-field evidence from Uganda reports a similar pattern when tokens allocated on a lottery are by default on the safe or risky choice [8]: more risky starting points yield less risk aversion in an identical choice set. In our setting, those who started seeing the insurance as more attractive, and have a less risky starting point, have a higher valuation of the risk reduction and are implicitly behaving as more risk-averse.

We also referenced the literature on choice architecture in our mention of the order effects in the conclusions. Lines 670-677 read:

> The attractiveness of the risk reduction is reinforced by one of our treatment outcomes: presenting the vouchers in descending order yields a higher take-up than the ascending order. We interpret this result in the light of an endowment effect for the insurance: the implicit risk reduction of taking valuable vouchers reduces the likelihood of opting for the cash payment when the voucher value decreases because this insurance becomes costly to forgo. Similar findings on the ordering of choices and take-up of insurance exist in the health sector [10, 22], and they call for choice architecture interventions to help people make more affordable insurance decisions.

6. The source of funding is disclosed, but there's no discussion on how it might have influenced the research. Include a statement about the independence of the research from the funding sources.

Thanks for pointing out. We received a similar comment as part of the Journal's requirements for the resubmission, asking us to update the Funding Statement and make explicit the independence of our research from funding sources. Moreover, we were also requested to update and remove the funding information from the Acknowledgments section and any other area of the manuscript. We thus update the Funding Statement as shown below:

> DC, CM and MR received funding from MinCiencias and the World Bank through the program "Inclusión productiva y social: programas y políticas para la promoción de una economía formal, código 60185, Alianza EFI." Grant number: FP44842-220-2018. <u>The funders had no role in study design, data collection and analysis, decision to publish, or preparation of the manuscript.</u>

We underlined the last sentence to remark, in this response, the statement regarding the independence of the research from the funding body.

7. Consent was obtained, but there's no mention of the process or any potential risks conveyed to participants. Elaborate on the ethical considerations, especially how participants were informed about the study's purpose and potential risks.

Thanks. We have elaborated on the ethical considerations by expanding the corresponding paragraph. Lines 245-258 read:

> The experimental protocol was approved by the Ethics Committee at Universidad del Rosario (approval code 459-CS295). <u>We obtained written consent from all participants after all the instructions were provided.</u> No minors were included in our study. <u>We informed the participants that the study was part of</u>

a project about the functioning of labor markets, funded by the Colombian Ministry of Science and implemented by a team of researchers from Universidad del Rosario and Universidad del Quindío. We emphasized our interest in understanding their preferences for cash or voucher payments for completing a task. We also informed participants that the collected information would be only used for academic purposes and that their payment would be made privately, minimizing any potential risk from the study. Our protocol also included a set of rules for conducting in-person sessions during the pandemic. We interacted in ventilated areas, with a maximum of fifteen participants, and mandatory use of hygienic face masks for participants and the research team during the entire activity.

The underlined text has been added to the resubmitted version. It responds to the Reviewer's request to emphasize how we informed participants about the study's purpose and its potential risks.

8. The statement about data availability is generic. Specify where the data can be accessed, whether it's an online repository or upon request, and ensure it's anonymized.

We apologize for this confusion. This information was provided at the beginning of the supplementary material and as part of PLoS ONE's submission questions, but not in the main text. We have amended this by adding one paragraph at the beginning of the Results section (lines 433-436), which reads:

> In this section, we present the descriptive results and then proceed with the regression analyses. We report here all the treatment variations and experimental sessions we have conducted for this research question. The data and script files to replicate all the tables and figures are available in the OSF repository https://osf.io/rp4xa/.

We now state that the data repository is in the Open Science Framework platform, in the abovementioned URL. Besides, we now make explicit that we report all the conducted treatment variations and experimental sessions.

9. Your study on the behavior of rural workers in Colombia provides valuable insights into the dynamics of rural labor markets. To further enrich your analysis and provide a broader context, I recommend referring to the following studies that delve into various aspects of rural labor dynamics in different regions:
• Zeeshan, Mohapatra, G., & Giri, A. K. (2022). How Farm Household Spends Their Non-farm Incomes in Rural India? Evidence from Longitudinal Data. The European Journal of Development Research, 34(4), 1967-1996.
• Santana, R. R. C., & Ristum, M. (2023). Child Labor in Families of Rural Workers: The Issue of Intergenerationality. Trends in Psychology, 1-16.
• Kolié, D., Van De Pas, R., Codjia, L., & Zurn, P. (2023). Increasing the availability of health workers in rural sub-Saharan Africa: a scoping review of rural pipeline programmes. Human Resources for Health, 21(1), 20.

Thank you for these suggestions. We have expanded the discussion on the related literature to mention that the mild acceptance of non-fungible payments may complement our understanding

of other rural household's mechanisms for coping with risk. We then mentioned non-farm enterprises (here, we cite Zeeshan et al., 2022—corresponding to [37]) and increases in the supply of labor (here, we cite Santana and Ristum, 2023—corresponding to [31]). Lines 99-105, at the end of the introduction, read:

> The mild attractiveness of non-fungible payments in rural contexts also contributes to the general understanding of the dynamics of rural labor markets, including income diversification in non-farm enterprises (e.g., small informal businesses [37]) and the increase in the household's labor supply to cover unanticipated shocks and anticipated liquidity shortages [18, 21].This supply response may also include child labor (that the household might see as intergenerational transmission of skills and values, according to qualitative information [31]).

We have also expanded our motivation for using a risk-reducing mechanism in exchange for non-fungibility: health and education shocks have persistent effects in rural contexts, and we can add the problem that the expansion of health services tends to bring undesired effects, as is documented in Kolié et al., (2022). Lines 29-34 read (the new text is underlined):

> We explore these preferences for accepting an in-kind payment in exchange for reducing risk exposure in a lab-in-the-field experiment with Colombian rural workers. <u>Risk reduction may be appealing in rural contexts, where shocks have more persistent effects on health and educational outcomes given the limited means to self-insure [2, 31], and the prevalence of unintended effects after the efforts to increase the supply of health services (e.g., see a review for sub-Saharan Africa in Kolié et al. [25])</u>.

Reviewer #2

The manuscript "Non-fungibility reduces the attractiveness of risk-reducing payments among rural workers: a lab-in-the-field experiment in Colombia" presents results of a series of field-based economic experiments to understand how rural people in Colombia value different payment types. The results are interesting, and mostly easy to understand. The quality of the writing is excellent, something that I greatly appreciate as it makes my work as a reviewer much easier. There are only small grammatical errors, some (but not all) of which I have flagged below. Thanks also for providing the version with line numbers.

I should say at this point that while I do use field experiments in rural areas of low- and middle-income countries, I do so mostly to test practical aspects of different incentive program implementations. My own research does not aim to test basic economic theory, something I have only basic familiarity with. When agreeing to review this paper I had not realized how much it was focused on fundamental theory, and I hope that at least one other reviewer of this paper is a real economist! I also note that some of the preferences I express in this review probably reflect that I come from a different disciplinary background.

Incidentally, I once did a pilot study in the same region you worked in (specifically the town of Filandia), with a demographically similar participant pool, and was impressed at how good at math they were. Perhaps it reflects particularly good instruction at schools there, but I've never seen rural workers anywhere else who figured out the optimal solutions to the game we were using so quickly. Thus, I would guess that many of your participants would have had a good grasp on the expected value of the different payment options. This probably has bearing on your results, particularly on where else they may be more or less relevant.

-Carl Salk

Dear Dr. Salk,

Many thanks for your suggestions and comments on our study. It was very encouraging to find in your report very thoughtful requests along with a friendly tone. We have taken all your comments into account, and we expect that you find our responses and amendments to the manuscript satisfactory.

Thank you for sharing your experience in Filandia. Speaking as one of the co-authors, I have the same impression of the numeracy skills of the rural population in Colombia: they tend to be rather high and usually uncorrelated with literacy skills. Based on a previous experience, a lab-in-the-field experiment with fishermen in the Caribbean, I got the impression that trade-oriented occupations with high intensity (i.e., catching and selling fish every day) trained their numeracy skills. In the case of these rural workers, the informal labor arrangements (i.e., not having a fixed contract with a given wage) may foster numeracy skills.

After reading this specific comment, the following details stem from a discussion among the research team, with the inputs of the researchers more aware of the local context: during harvest time, rural workers are paid based on the amount of coffee collected. This quantity is weighed daily, at the end of each work journey, but they get paid weekly. Besides, they live on the farm on the days of harvest. There, they pay for their meals and additional food expenditures, so this is

deducted from their weekly wage. Typically, the workers carry this information as mental accounts, whereas the farm managers (who are in charge of paying them), keep the written accounting with each worker.

This story reinforces the mechanism where numeracy skills may be strengthened by the type of compensation arrangements the workers face. Something that is worth exploring in future studies. In fact, we considered making this argument in the paper, but we realized that it would be unsubstantiated. Definitely, it would be a good idea to include a couple of numeracy questions in future instruments using lab-in-the-field experiments or directly delve into compensation arrangements and numeracy skills.

Nevertheless, after reading this comment we believe that it was important to remark that instructions appeared to be properly understood, according to the increasing take-up as the voucher value increases. Hence, in our results section, we added the following statement (lines 458-460):

> From a more general perspective, the take-up is increasing in V for the rural workers in panels (a), (b), and (d). We interpret this pattern as a basic validation check that the instructions were properly understood.

In the rest of this response letter, you will find our responses in blue and any quotation from the manuscript will be indented and in a different font.

Again, thanks for your comments. We believe that they helped us produce a better version of our manuscript.

Specific comments:

Line 7: “developing context” – is a word missing here? Developing country?

Thank you. We wrote this sentence again for clarity. It now reads:

> In the context of developing countries, such violations…

L41-65: This seems to be a bit too much results for so early in the intro. Perhaps summarize this as a few sentences at the very end of the intro?

We followed your suggestion and restructured the introduction. Instead of presenting in the same paragraph elements of the design and results (from the rural workers and the students), we now introduce details of our lab-in-the-field design and implementation in a couple of paragraphs, and then we dedicate one paragraph to a more compact preview of results for the rural workers. This paragraph (lines 65-73) reads:

> As a preview of our findings, the voucher take-up goes from 32% for the least generous voucher to 56% for the most generous voucher. We did not find differences in the voucher take-up depending on whether the show-up fee was delivered in cash or as a voucher. By contrast, we found weak evidence that presenting the vouchers in descending order (i.e., showing in the first decision the most generous voucher) increased the voucher take-up. We conjecture that, by facing the most attractive voucher first,

participants experienced an ``endowment effect'' [24] with the insurance (i.e., that they place more value on the insurance by the fact of having it), increasing the cash premium required to forgo the lower probability of a loss.}

This paragraph is followed by another one (lines 74-83), in which we introduce the results from the sample of students from a more comparative perspective. It reads:

> To put the voucher acceptability in perspective, we implemented the same instrument with a sample of 69 university students from the region's main city, Armenia. Non-fungibility should be even starker in this sample since students in this region are rarely in charge of grocery shopping at their homes. Indeed, we find that their voucher take-up goes from 16% to 33%, from the least to the most generous voucher.
>
> These numbers are considerably below the voucher take-up in the rural sample (e.g., the workers' take-up for the least generous voucher matches the students' take-up for the most generous voucher). Unlike rural workers, students appear to be influenced by the redemption costs: they were more likely to select the voucher if their show-up fee was delivered as a voucher as well.

L55: Is a word missing after "incur"?

According to our reading, there was no missing word. However, your comment pointed out some readability problems in this sentence, so we rewrote this sentence (lines 53-55) as:

> If participants perceive that the voucher has a positive redemption cost, those receiving the show-up fee in a voucher would be more likely to select this payment for completing the real-effort task because they already need to incur this cost.

L92: Please also provide the approximate value of the payout in US dollars.

Thanks. We have addressed this comment with three modifications to our text:

- We added the following sentence (see lines 111-113) the first time we mentioned the COP currency:

  > The average exchange rate between July and August 2021, the months in which fieldwork took place, was 3,877 COP for 1 USD.

- We report the approximate value of the payout for the cash option in parenthesis. It reads:

  > *Cash*: Receive 30 kCOP (7.74 USD) in cash for task completion. Face a **20%** chance of losing 20 kCOP (5.16 USD).

- We opted not to add this information to the *voucher* option, in the other bullet point, since V is variable and may be confusing. However, we added the range of values for V in USD after listing the set in kCOP. Lines 125-126 read:

  > Participants took four decisions with different voucher values given by $V \in \{25; 28; 30; 34\}$ [kCOP] (in USD, V goes from 6.45 to 8.77).

L126: Change to "Panel A in Table 1 summarizes the comparisons..."

We have followed your suggestion. Thanks!

L165: Perhaps I am missing something, but I thought there were only two choices (i.e. those shown from L92-95). Please clarify! Ok, on going back to L100, I think I understand this now – it seems that participants were asked to make 4 different decisions. This could be made more clear up front.

We appreciate this suggestion that we addressed in two ways. First, we borrow your wording and now call them "decisions" and not "choices", as the latter term can also refer to the two payment options. We corrected the manuscript accordingly to talk about four decisions, and in each decision there were two choices: cash or voucher. Second, we substitute "They have to choose one of two options" with "They had to pick one of two choices" (see lines 115-116).

L168: "play" → "plays"

Corrected. Thank you!

L193 "draw" → "drew"

Corrected. Thank you!

L196 "explain" → "explained." There is a lot of switching between present and past tense in this section. I would recommend past tense, but whatever you choose, please be consistent!

Thanks for this suggestion, and our apologies for not taking more care of this consistency in our initially submitted manuscript. We have now reviewed the entire paper looking for these inconsistencies and eliminated all the incongruencies within a given paragraph, and also improved the changes between present and past tense when the paragraphs were having similar purposes (e.g., describing aspects of the task, or summarizing results).

Regarding the specific section on the implementation procedure, we amended the mixed past and present tenses, sticking to the latter as you suggested.

L199: Delete "on"

Corrected. Thank you!

L261-262: Change "Quindío is the smallest Department in total extension" to " Quindío is the smallest department by area."

Thank you. We have corrected this sentence according to your suggestion.

L282 (and 285): "University" does not need to be capitalized since you are not using the word as part of the specific name of the university here.

Thank you. We have amended this mistake in all the four relevant cases.

L288: The first sentence here is unnecessary.

This sentence has been deleted.

L294: Change “government’s” to “government”

Thanks. We have modified all the similar sentences to “government subsidy.”

L295: “were” → “was”

Corrected. Thank you!

L325-7: Names of the fields of study do not need to be capitalized.

Corrected. Thank you!

L333, etc.: Percentages can generally be written out as numbers.

Thank you. We have changed all the percentages that were not at the beginning of a sentence to numbers.

L337-340 (and many other similar passages): Move to discussion section.

We have adjusted the manuscript, so it now includes a discussion section. We moved here all the passages that involved conjectures made right after presenting the results. We wrote from scratch this discussion around two central points: (i) what we learned from the comparison between rural workers and students, and (ii) some comments on the statistically significant variables in the exploratory analysis.

Since this is an entire new section we omit it from this response letter to not make it too large.

L347 (and elsewhere): Just need to say “incur” rather than “incur in.”

All four uses of “incur” have been corrected. Thanks!

Table 1: I realize tables like these are standard for economists, but in many other disciplines a series of barplots would be used, something I generally find easier to interpret.

Thanks for this suggestion. We created Figure 2, which includes four panels. A top panel (a) with the bar plot comparison between samples. Then, we leverage on the colors to put on the left side the between-treatment comparisons for the rural sample in panels (b) and (d), and on the right side, in panels (c) and (e), the corresponding comparisons for the sample of students. We appreciate this comment as the Reviewer is correct in that it improves the ease of comparability of our main results. Since the main results are now displayed with a figure, we have also edited the accompanying written description of these results.

Regarding the **old** Table 1, Panels B and C, with the statistical comparisons, were moved to the new Table A.4 in the appendix. They are now called Panels I and II to avoid confusions with the names of panels in Figure 2. Panel A was moved to the Experimental Design section, which now constitutes the main part of the **new** Table 1, to which we added some extra clarifications on the theoretical predictions.

L382: Should say "participants."

Corrected. Thank you!

Figure 2: Again this is probably a disciplinary difference, but the stair-stepped graphs are odd to me – I would prefer a standard line graph with individual data points shown using symbols. This would also reduce the overlap of the two lines. Also, I find the >34 category always being 100% to be a bit misleading (and hard to even see as presented since the black dashed lines always disappear behind the gray lines – if you want to keep the stair-stepped look, you could at least make the black dashed lines thicker so they stick out behind the gray lines). There may be participants who always prefer cash, no matter how large the voucher value is.

Thank you for your comment. It led us to think about how to convey the usefulness of the stair-stepped graph as part of our results. First, we start this subsection with a clearer definition of the minimum acceptable voucher, the variable plotted in old Figure 2 (now Figure 3). Lines 476-482 read:

> We define the *minimum acceptable voucher* as the lowest value of V for which the participant selected the voucher instead of the cash. This aggregated measure collapses the four decisions into a single observation per participant. Our *minimum acceptable voucher* can be interpreted as a willingness to accept (the voucher instead of a cash payment), with a caveat: preference reversals (e.g., taking the voucher for $V = 28$ and $V = 34$ but the cash for $V = 30$) are treated as mistakes that do not affect the lowest switching point toward the voucher.

We underlined the new elements in this description that are most relevant in this explanation: the minimum acceptable voucher is a measure of "willingness to accept" (the voucher instead of cash), or WTA. For the WTA, the cumulative distribution is helpful to illustrate the total acceptance of the voucher for a given value, V. We now explain this in lines 485-488, which read:

> Figure 2 plots the cumulative distribution of the minimum acceptable voucher. We use a cumulative distribution given its interpretation as a willingness to accept: we obtain an idea of the aggregate voucher acceptance for each of the four values V in the horizontal axis (i.e., the ``steps'' of the staircase).

We also acknowledge that the category "$V > 34$" might be hard to interpret, so we replaced it with "unk(nown)" in Figure 3, and provided the following explanation in lines 488-491:

> Nonetheless, we do not know the minimum voucher value for those participants who always preferred the cash (accounting for 35% of the combined samples, or 99 out of 283), so the last vertical step in the cumulative distribution is marked as *unknown*.

Finally, we adjusted Figure 3, so it has some continuity with Figure 2 in terms of colors and legends. We were also careful to put behind the dark continuous lines and in front of the light and dashed lines. By doing this, we guaranteed that none of the lines fully disappeared behind the other, and instead, we got a dashed “dark-light” pattern.

Table 2: This table is confusing in that (and I’m still not 100% certain about this) a negative coefficient in the tobit analysis indicates the same thing that a value between 0 and 1 means for the ordered logit analysis. At first glance, I thought you had different results since one set of coefficients was negative and the other positive, but then I noticed that one was an odds ratio. This difference needs to be highlighted to prevent others from getting confused like I did.

You are right about the need for early clarification on how to interpret the coefficients in this table, thanks. To amend this issue, we have added a paragraph before the explanation of the results reported in Table 2. Lines 512-525 read:

> Whereas the tobit model has a more practical interpretation, the ordered logit accounts better for the large share of participants that always preferred the cash payment. Tobit coefficients can be interpreted as in an OLS model, but the linear effect is on the uncensored latent variable (i.e., as if we would have identified minimum acceptable vouchers beyond $V = 34$, not the observed outcome. The coefficients in an ordered logit model are more convoluted to interpret because they are directly estimated as log odds. To interpret them in odds, we need to apply an exponential transformation. The positive coefficients will be transformed into odds larger than 1, indicating a higher probability of selecting highly ranked categories. By contrast, the negative coefficients will be transformed into odds between 0 and 1. In our specific case, an odd below one will indicate that an increase in the independent (explanatory) variable would reduce the selection of a higher switching valuation, meaning that vouchers are preferable as a lower V is required to switch from cash to voucher payment.

Besides, we rewrote the description of the results from the ordered logit coefficients in Table 2. Lines 536-540 read:

> For the ordered logit, the reported coefficients in odd ratios fall below one. Hence, they indicate a lower probability of selecting a higher category (i.e., a larger switching value V that makes a voucher preferable to cash). Thus, presenting the vouchers in descending order makes *less* necessary a larger voucher payment to induce this switch from cash.

L452: Change “neither” to “either.”

Corrected. Thank you!

L472-3: Change “incurring” to “engaging.”

Corrected. Thank you!

L487: Change “worst” to “worse.”

Corrected. Thank you!
