# Supplementary material for: Are non-fungible payments attractive when they reduce risk exposure? Evidence from Colombia
Source: PLoS One. 2024 Jan 2;19(1):e0296299. doi: 10.1371/journal.pone.0296299 (PMC10760666; doi:10.1371/journal.pone.0296299)
Supplement: Attachment — Submitted filename: Response Letter_RR2.pdf [file pone.0296299.s003.pdf]

Comments from Reviewer #2

Thank you for your revisions. This manuscript is now in excellent shape and I enthusiastically recommend its acceptance. Below I have noted some minor points that could further improve the clarity of the writing.

Best regards,

Carl Salk

Dear Dr. Salk,

Thank you for your very positive and encouraging assessment of our manuscript. We appreciate the time and effort you dedicated to providing very detailed comments that helped us polish the writing.

Abstract (and elsewhere): The word "uptake" would sound more natural than "take-up."

Thanks. We have changed all our mentions of "take-up" to "uptake," including the appendix.

L85: Change to "developing-country context"

Done.

L138: Change "less" to "least."

Amended. Thanks!

L186-187: Change to "the municipalities' capitals tend to be relatively compact." In general (and I can't believe I didn't catch this in the first review), the phrase "municipality's head" sounds like a person who is the chief executive of the municipality. I think you are talking about the town where the municipal government is based. There is probably no perfect translation for this in English, but "capital" is my best bet. "Market town" or "main town" may also work. I note this phrase appears several times in the manuscript.

This is a good point, and we struggle a bit looking for how to translate this more conglomerated area of the municipalities (*cabecera* would be the name in Spanish). We are happy to follow your suggestion, so we now call it "municipality's capital" throughout the text.

Besides, we added one more clarification when we first introduced the term. Lines 190-194 read:

> Most of (if not all) the participants recognized the name of the supermarket, since the municipalities' capital (or *cabecera*, which the reader may visualize as the main town, though hereafter we will refer to this more aggregated area of the municipality as its capital) tend to be relatively small in extension.

L330: I think "list" would be clearer than "timing" here.

Thank you. We agree that it reads better with "list." We changed the parenthesis to:

> (steps *v* to *ix* in the list above)

L350: “Quindio is the smallest mainland Colombian department by area.”

Thanks for your help. It definitely sounds better and we can remove the initial sentence (“Excluding the Colombian insular zone”).

L380: “In-person” would sound more natural than “in-presence.”

Amended. Thanks!

L388: Change “of” to “as.”

Amended. Thanks!

L404: Change “during” to “for.”

Amended. Thanks!

Figure 2: I think the y-axes should be labeled “proportion” rather than “%.” Or the numbers on the scale could be multiplied by 100 to fix this.

We have adjusted the Figure according to your suggestion. Thanks. We adjusted the figure’s caption as well. We show below three out of the five panels.

Fig 2. Voucher uptake (as proportions) in the rural and students’ sample (by treatment).

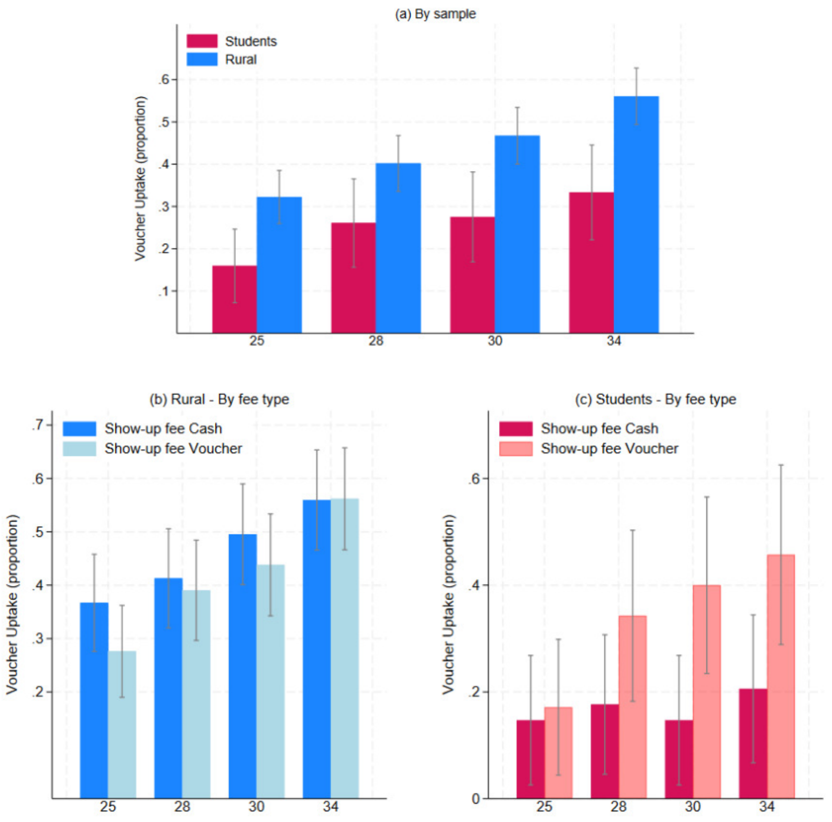

L485: I believe this should say “Figure 3,” not “Figure 2.”

Our apologies. Thanks for spotting this mistake! It has been amended.

L487: “On” would sound more natural than “in.”

Thanks. We agree with your suggestion and adjusted accordingly.

L560: Change “make” to “makes.”

Our apologies. This has been amended.

L562: “Tobit” does not need to be capitalized.

You are right. We changed it. Thanks!

L565: Change “in” to “by.”

Amended. Thanks!
